# Electronic and Thermal Properties of Graphene

**DOI:** 10.3390/nano10050926

**Published:** 2020-05-11

**Authors:** Kyong Yop Rhee

**Affiliations:** Department of Mechanical Engineering, College of Engineering, Kyung Hee University, Yongin 446-701, Korea; rheeky@khu.ac.kr

Recently, the development of nanotechnology has bloomed in numerous industries. In this regard, graphene, a two-dimensional carbon nanomaterial, has been extensively researched, due to its high academic interests and commercial potential. Due to its unique structure and outstanding material properties, the research on graphene has enabled substantial progress in the development of electronics devices, thermoelectric appliances, optical devices, sensors, and energy harvesting applications. In addition, the mechanical properties, such as young modulus (1TPa), intrinsic strength (130 GPa), high stiffness, and fracture strain, make graphene a promising candidate for industries like nanocomposite coatings, paints, and bioapplications [1]. The development of graphene and graphene-based nanocomposites is always under intensive research, to provide the foundations for next generation devices. In this framework, the present special issue explored recent advances in the graphene based devices and their applications in next generation electronics. Here, we have discussed remarkable electronic and thermal properties of graphene such as Dirac fermions, high electrical conductivity, high seebeck coefficient, the quantum hall effect and thermoelectric effects [2]. This special issue compiles 19 articles dedicated to thermal, electrical and thermoelectric properties of graphene: 14 research articles and five review articles.

Graphene shows numerous novel and inimitable physical properties, such as the anomalous quantum hall effect, the ambipolar electric field effect, Klein tunneling, and ballistic transport, due to its exclusive crystal and electronic structure. However, the band gap characteristics of graphene limits its applications and commercial interests. The properties of graphene can be tuned by altering its nanostructure in terms of boundary configuration, structural defects, chemical doping, and the formation of heterogeneous structures to meet the demands of the electronic, photo thermal, thermoelectric and photoelectric fields [3]. High energy density and low cost are the prerequisite conditions for next generation transportation and grid storage. Lithium sulfur batteries are widely popular in this respect, however, due to their low active material utilization and poor life cycle, Hearin Jo et al. [4] use carbon coated separators with different ratios of carbon black and vapor grown carbon fibers to inhibit the migration of the soluble polysulfide, preventing it from reaching to the Li metal surface. Hong et al. [5] prepared carbon foam from carboxymethyl cellulose and compared enhancement in the thermal conductivity of neat carbon foam (CF), and carbon foam with Ag (CF-Ag), Al (CF-Al), CNT (CF-CNT), and graphene (CF-G). Their studies showed higher thermal conductivities of CF-Ag, CF-Al, CF-CNT, and CF-G, compared to CF. Among all the CF, the phase change temperature is the fastest for CF-G. In addition, the SEM analysis showed effective impregnation of erythritol into the pores of carbon foams, which minimized the latent heat loss during thermal cycling. Therefore, these CF impregnated with erythritol represents promising material for thermal energy storage applications. Jhao Yi Wu et al. [6] reported a facile process to synthesize few layer graphene (FLG) from graphite by a liquid exfoliation process. The similar D/G ratio of FLG and graphite demonstrates generation of very few structural defects. The FLG/polyvinylidene difluoride thin films showed superior electrical conductivity with great flexibility and mechanical strength under bending conditions, and have immense potential in conductive adhesive applications. The manuscript by Maoyuan Li [7] and Huaipeng Wang [1] discussed molecular dynamics simulations (MDS) to study the mechanical properties of graphene and carbon honeycomb (CHC). Maoyuan Li et al. investigated the effects of temperature, strain rate, and defects in the mechanical properties of graphene. Their results indicate a reduction in the Young modulus, fracture strength, and fracture strain with increases in the temperature. The existence of defects in graphene hampers the mechanical properties significantly, due to local stress concentration points, whereas the thermal conductivity showed a low temperature dependent behavior. In a similar study, Huaipeng Wang et al. presents an interesting insight between the covalent bonds of hinge atoms of CHC and their plastic behavior.

The special issue is completed with review papers [2,3,8,9], which compile recent research findings on the thermal, electrical, and thermoelectric properties of graphene and its composites. In addition, the application of graphene in the development of electrochemical biosensors was also discussed. Chao Lv et al. [10] discussed recent developments and the potential of graphene-based humidity sensors in their review paper. 

In summary, this special issue of nanomaterials entitled *“Electronic and Thermal Properties of Graphene”* compiles a series of original research articles and recent review papers to provide depth information on the development of graphene based next generation devices. We hope that this special issue will provide an interesting insight into the latest perspectives in this rapidly evolving industry.
